# Application of Inertial Microfluidics for Isolation and Removal of Round Spermatids from a Spermatogenic Cell Sample to Assist *In-Vitro* Human Spermatogenesis

**DOI:** 10.3390/mi16050500

**Published:** 2025-04-25

**Authors:** Sabin Nepal, Joey Casalini, Alex Jafek, Bruce Gale

**Affiliations:** 1 Department of Mechanical Engineering, University of Utah, Salt Lake City, UT 84112, USA; sabinnepal@hotmail.com; 2 Paterna Biosciences Inc., Salt Lake City, UT 84119, USA; joeycasalini@gmail.com (J.C.); a.jafek@gmail.com (A.J.)

**Keywords:** inertial microfluidics, particle separation, cell separation, *in-vitro* spermatogenesis, dean flow, human germ cells

## Abstract

*In-vitro* spermatogenesis holds great potential in addressing male infertility, yet one of the main challenges is separating round spermatids from other germ cells in spermatogonial stem cell cultures. STA-PUT, a method based on velocity sedimentation, has been extensively tested for this application. Though somewhat effective, it requires bulky, expensive equipment and significant time. In contrast, the method of inertial microfluidics offers a compact, cost-effective, and faster alternative. In this study, we designed, fabricated, and tested a microfluidic spiral channel for isolating round spermatids and purifying spermatogenic cells. A commercially available spiral device close to the calculated specifications was tested for rapid prototyping, achieving 79% purity for non-spermatid cells in a single pass, with ability to achieve higher purity through repeated passes. However, the commercial device’s narrow outlets caused clogging, prompting the fabrication of a custom polydimethylsiloxane device matching the calculated specifications. This custom device demonstrated significant improvements, achieving 86% purity in a single pass compared to STA-PUT’s 38%, and that without any clogging issues. Further purification could be attained by repeated passes, as shown in earlier studies. This work underscores the efficacy of inertial microfluidics for efficient, high-purity cell separation, with the potential to revolutionize workflows in *in-vitro* spermatogenesis research.

## 1. Introduction

An estimated 15–20% of couples struggle with some form of infertility globally, and male infertility factors are thought to produce half of that number [1]. Most male infertility cases stem from a deficiency in the motile sperm cells in the ejaculate, a condition known as azoospermia. The most severe form of azoospermia is non-obstructive azoospermia (NOA), which occurs when the ejaculate has no sperm cells and there is minimal to no sperm production taking place within the seminiferous tubules [2]. The causes of these conditions are some forms of damaged testicular microenvironment [3,4]. The possibility of *in-vitro* spermatogenesis (IVS) opens an avenue to solve the problem of yielding enough healthy and viable cells by providing a healthy microenvironment outside the body. Even though there has been some progress in mice and some other animals, IVS research in the case of humans is still in its nascent stage. A detailed review of the history of IVS can be found here [5]. The researchers in this field face a significant challenge of removing round spermatids from other germ cells before culture. Typically, the goal is to convert primary spermatocytes into round spermatids. However, if round spermatids are already present in the sample, it introduces a skew in the results and thus complicating further analysis and inhibiting effective research. A single-cell suspension prepared from a digested testicular tissue contains all phases of germ cells, thus requiring the separation of round spermatids and purification of the other germ cells.

Significant work on a wide variety of cell separation techniques can be found in the literature [6], and multiple comprehensive reviews of the application of micro and nanotechnologies for particle separations are available [7,8]. In general, most of these techniques either have low cell recovery and throughput and are hard to automate or they are too complicated and expensive. Often, they rely on labels, which are not allowed when used as human infertility treatments. Additionally, many approaches require external force fields, making them impractical for commercialization. In this particular case, there has been no method demonstrated for separating these cells. The technique explored and reported the most for separating these spermatogenic cells, albeit non-human, is called STA-PUT [9,10,11,12]. The method is based on velocity sedimentation and offers the benefit of high yield. However, the method does not result in high-purity cell fractions, requires large, expensive apparatus, and the process requires hours for a single run [12]. Unfortunately, high cell purity is of primary importance for application in *in-vitro* spermatogenesis, while yield is a lower priority. In our experiments, the method was able to increase the purity of cells other than round spermatids to about 38%. These results are not ideal, so there is a strong desire for a commercially scalable low-cost system that efficiently separates testicular cells without labels and has a high recovery and throughput.

Microfluidics, the study of fluid behavior and its interaction with particles on a microscale, offers an avenue for separating particles based on their properties [13]. Principally, microfluidic particle separation techniques can be divided into the following two types: (i) active: methods that utilize external force fields, and (ii) passive: methods that utilize flow physics and channel geometry [14,15]. Since active methods rely on external force fields, they require external systems to drive particle separation. Since we wanted to develop a simple, label-free system that could effectively separate the cells at low cost, passive methods were of more interest to us. Techniques like deterministic lateral displacement (DLD), pinched flow fractionation (PFF), microfiltration, and microfluidic hydrodynamics fall under the heading of passive methods. Among these passive methods, microfluidic hydrodynamics offer the possibility of high throughput cell separation compared to other techniques [14]. However, these techniques are also very sensitive to flow disturbances and can result in high pressure drop in the channel at higher flow rates.

Inertial microfluidics is a hydrodynamic technique for particle separation, where a curved channel is used to result in a secondary lateral flow that can separate particles based on shape and size. Our lab has utilized inertial microfluidics for passive and label-free particle separations in the past for different cell separations, including the separation of sperm and blood cells [16,17,18,19]. The method has also been reported to have high cell viability [18]. Among other applications, this same principle has also been implemented to separate circulating tumor cells (CTCs) [20] and cancer cells from whole blood [21]. While they still have some drawbacks like high sensitivity to flow disturbances and a high pressure drop along the channel, a spiral channel designed to the correct specifications and operated at a proper flow rate can focus particles of different shapes and sizes to specific locations along the channel width. The primary spermatocytes, on average, have a 15 μm diameter, while the average diameter for the round spermatids is 6 μm, thus opening an avenue for a size-based separation. The separated particles can then be extracted into separate streams with a strategically designed outlet.

While inertial microfluidics has seen many implementations for the isolation of sperm, it has not yet been applied and studied for the isolation of sperm precursors. So, in this work, we apply the empirical equations governing particle behavior in a microfluidic spiral channel to design and fabricate a device that removes round spermatids from a mix of digested human testicular cells. First, we model and calculate the required design parameters. Second, we explore a commercially available device that closely matches the calculated design requirements. Lastly, we fabricate and test a device with improvements specific to the cell types for an increased performance. The devices have low fabrication and operating costs, operate without cell labeling, and result in a substantially higher cell purity while being much faster than STA-PUT.

## 2. Materials and Methods

### 2.1. Spiral Channel Design

**Figure 1:**
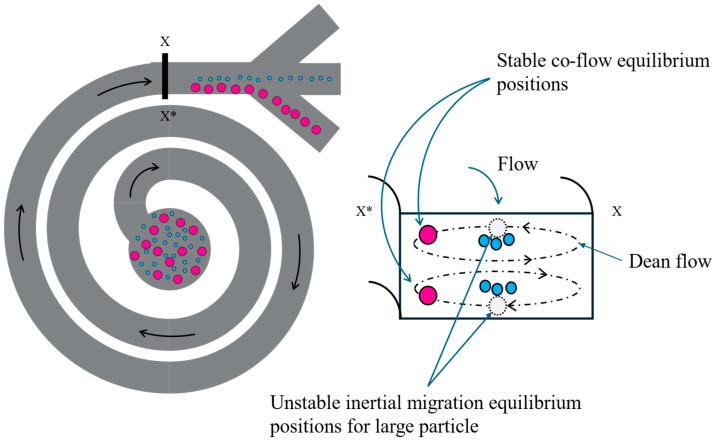
Flow focusing and separation in a curved channel with a rectangular cross-section. In a straight rectangular channel, the net inertial lift force causes particles to focus toward the wider channel walls, as the dotted circles represent. However, once the channel is curved, a secondary flow called Dean flow develops due to the outward acceleration of the fluidic mass in the channel center. Depending on the characteristic features of the channel, these vortices displace the largest particles located farthest from the center towards new equilibrium positions, as shown. In contrast, the smaller particles would focus closer toward the vertical center of the channel.

Particle-focusing behavior in a spiral channel has been well established in terms of empirical equations [22,23,24]. To recapitulate, when particles of different sizes are introduced into a straight channel with a rectangular cross section, the net inertial force causes the particles to focus along the channel center and towards the longer faces. As shown in Figure 1, when curvature is introduced in the channel, acceleration along the radial direction of the spiral causes the fluid at the center to flow in the outward direction and form a secondary flow in the channel. Based on conservation of mass, the space at the center is now occupied by the fluid from the top and bottom portion of the channel cross-section. Consequently, these lateral movements of fluid give rise to Dean vortices. If a particle is introduced into the flow, on top of the net inertial lift force that pushes the particle away from the center, the particle also experiences force due to the lateral Dean vortices. The Dean drag forces that each particle experiences depend on its shape and size, thus resulting in their unique final equilibrium position in the channel based on the balance of the net inertial lift force and Dean drag force.

Overall, the net inertial lift force causes the particles to focus, and the Dean vortices move them along the radial direction, causing the focused particle streams of different shapes and sizes to shift to new locations along the channel cross-section. A prior investigation has documented that achieving particle focusing in microchannels requires a channel block ratio (*β*) above 0.07, wherein *β* represents the ratio of particle diameter to channel height [22].

Table 1 compiles the parameters, dimensionless numbers, and equations that characterize particle separation in inertial and Dean flow.

Smaller particles have more stringent requirements for inertial focusing in a spiral channel. As long as a channel is designed with the smallest particle size in mind, larger sized particles should be able to focus and separate. Based on this theoretical principle, we calculated an appropriate channel design by first considering a successful inertial focusing for the smallest particle size. The smallest cells of interest in this work were round spermatids with an average cell diameter of 6 μm. Considering all the conditions that dictate particle focusing, we calculated that a spiral channel with a cross section of 300 μm × 80 μm with a radius of curvature of 7 mm would result in the focusing and separation of 6 μm particles. To validate these calculations, devices with cross sections of 300 μm × 55 μm, 300 μm × 62 μm, and 300 μm × 73 μm were also fabricated and tested at different flowrates. Finally, a flow rate of 1.5 mL/min was selected based on the resulting forces for particle separation, while keeping in mind the device’s integrity. The results from these calculations and tests can be found in the Supplementary Materials (Figures S1 and S2 and Table S1).

### 2.2. Microfluidic Devices

First, a commercial poly (methyl methacrylate) (PMMA) device was acquired and tested for simplicity and rapid testing. We found a rigid general-purpose commercial microfluidic spiral channel (microfluidic ChipShop, Fluidic 382) with four spiral sorting units. Among the four units, the sorting unit 2′s parameters were close to our requirements. The device was made from PMMA, thus was rigid, and the specification sheet of the device mentions that the unit’s cross-section is 300 μm × 80 μm, and the channel length is 166 mm. The design incorporates eight equally sized outlets with a width of about 90 μm.

**Figure 2:**
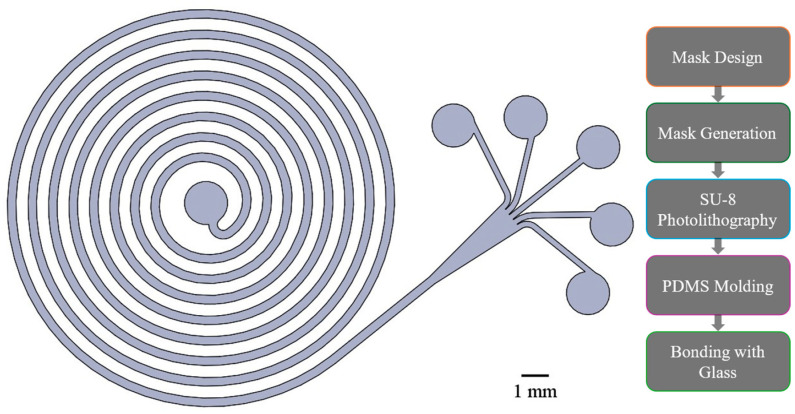
CAD design of the spiral channel based on the empirical model and the device fabrication protocol. A photomask was fabricated based on the design and was used to create an SU-8 mold on a silicon wafer. The mold was used to transfer the pattern onto a PDMS, which was then bonded to a glass with corona treatment.

Second, we fabricated an in-house device to match the calculated design specifications precisely. To maintain biocompatibility, the device was fabricated by molding PDMS (Sylgard 184, Dow Corning, Midland, MI, USA) on an SU-8 (SU-8 3035, Microchem, Westborough, MA, USA) mold. First, a photomask was developed based on the CAD design of the spiral channel (Figure 2). Then, a 100 mm (4 in.) silicon wafer was used to create the SU-8 mold in a cleanroom environment, following the manufacturer’s instructions. Once the mold was prepared and ready to use, 50 mL of uncured PDMS base was blended with 5 mL of curing agent. The mix was poured over the mold after ensuring it did not contain air bubbles. The mold was then subjected to a temperature of 73 °C for 60 min. Once crosslinked and cured, the PDMS was carefully peeled off from the mold, and the center inlet and the five outlet holes were punched into the PDMS using a 1.5 mm diameter punching tool.

Next, the channel-side surface of the PDMS was meticulously cleaned and plasma-bonded to a clinical-grade glass slide (Thomas 6686M20, Thomas Scientific, Swedesboro, NJ, USA), forming a closed channel. Lastly, 1.5 mm silicone tubes were affixed to the inlet and outlets to facilitate the injection and collection of test samples.

### 2.3. Experimental Modeling Using Spherical Beads

Before conducting tests with biological samples, a series of experiments were carried out in the PDMS device using 6 μm (blue), 10 μm (green), and 15 μm (red) fluorescent polystyrene beads to visualize the flow-focusing and separation within the spirals and validate the empirical model. The PDMS device was selected for validation since it would allow us to incorporate the desired outlet design into the test. The beads were mixed in equal amounts and diluted in phosphate-buffered saline (PBS), resulting in a total concentration of around 250,000 beads/mL. This concentration was selected based on multiple preliminary tests through the device at concentrations ranging from 150,000 beads/mL–300,000 beads/mL. The 250,000 beads/mL resulted in a balance between throughput and particle–particle interaction, since higher particle–particle interaction can otherwise defocus the focused particles. The device was primed using PBS, and any air bubbles present within the device were removed under careful observation under an inverted microscope, Invitrogen EVOS M7000 (Thermo Fisher Scientific Inc., Waltham, MA, USA). If air bubbles are left intact within the channel, especially towards the outlets, they can disrupt the flow focusing and locations of the particles. Once the device was ready, a 5 mL syringe was used to draw 5 mL of the prepared bead sample. The syringe was placed on a syringe pump and connected to the device’s center inlet. On top of the infusion through the inlet, withdrawing at the same total flow rate from the outlets ensures an equal flow rate in each outlet. Thus, four other syringes were connected to the four inner outlets and placed on a larger pump capable of operating eight syringes. The outermost outlet was left in the atmosphere to adjust for discrepancies between the infusing and withdrawing flow rates; its output would be collected in a tube.

Once the system was ready, the device was placed under the microscope for fluorescent imaging using the Invitrogen EVOS FL Auto 2 Imaging System. The infusing syringe pump was run at a flow rate of 1.5 mL/min, and the withdrawing syringe pump was run at a flow rate of 0.3 mL/min. The live feed from the microscope was observed on the interface. Once the flow-focusing stabilized for all three particle sizes, stacks of images were captured at multiple locations on the device for quantitative analysis. The outputs collected at the outlets were centrifuged at 300× *g* for 5 min, and the supernatants were removed to concentrate the collected beads. Fluorescent images of each of the outputs were captured for a qualitative analysis. Additionally, each centrifuged output was pipetted into three wells of a 384-well plate separately and was imaged and counted using a size-based image analysis.

### 2.4. Separation of Spermatogenic Cells

The tissue samples used in this work were obtained and used under Donor Connect protocol #F-RS-1. Fresh human testicular tissues were digested using standard sequential collagenase IV and trypsin tissue dissociations. In brief, human testicular tissues were gently spread using razor blades, exposing the seminiferous tubules, digested using 1 × collagenase IV (C4-22-1G, Sigma-Aldrich, Darmstadt, Germany) for 5 min at 37 °C, washed with 1 × PBS, and digested again using 0.25% Trypsin (25200056, Thermo Fisher Scientific, Waltham, MA, USA) for 30 min at 37 °C to form a single cell suspension.

After the sample preparation, 5 mL of the sample was drawn into a 5 mL clinical syringe. The device was primed with cell media, and air bubbles present inside the device were removed with careful observation under the microscope. As in the case of the beads, four outlet syringes were connected to the inner four outlets of the device, and the outermost outlet was left out in the atmosphere. The inlet and outlet syringes were put on their respective syringe pumps. The inlet syringe pump was run at a flow rate of 1.5 mL/min, and the outlet pump was run at a flow rate of 0.3 mL/min to keep the overall flow rate the same. This allowed us to capture equal volumes through all five outlets. Output from the fifth outermost outlet was collected in a tube.

The equal volume of collected outputs from each outlet was centrifuged at 300× *g* for 5 min and concentrated for imaging and analysis. The appropriately concentrated output samples from each outlet were pipetted into a 384-well plate and centrifugated at 200× *g* for 1 min to allow all cells to settle. Immediately after centrifugation, bright field images of all cells in each well were collected using the EVOS M7000 Microscope (AMF7000, Thermo Fisher Scientific, Waltham, MA, USA). Bright-field images were segmented using Cellpose to measure the cell diameter of collected cells. Segmented objects were then measured in ImageJ (version 1.54p).

After the bright field images were collected, the cells were incubated overnight at 35 °C. The following morning, the cells were fixed and stained using standard methods. In brief, the cells were fixed with 4% PFA (XX) for 5 min and washed 3 times with 1 × PBS. Dead cells present in the output degrade and are washed away during the cell fixing and washing, so only live cells remain for the staining protocol that followed. Cells were then permeabilized with 0.1% Triton-X for 10 min at room temperature and washed 3 times with 1 × PBS. The cells were then incubated in a blocking buffer (PI37536, Thermo Fisher Scientific, Waltham, MA, USA) for 1 h. Without washing, cells were stained with Rabbit anti-ACRV1 antibody (14040-1-AP, Proteintech Group, Rosemont, IL, USA) at 1:200 in blocking buffer at room temperature for 1 h and washed 3 times with 1 × PBS. Cells were then stained with donkey anti-rabbit 594 (A21207, Thermo Fisher Scientific, Waltham, MA, USA) at 1:800 and Hoechst 33342 (H3570, Invitrogen, Waltham, MA, USA) for 1 h at room temp and washed 3 times with 1 × PBS.

Fluorescent images of ACRV1 stained cells were acquired with the EVOS M7000 microscope (ThermoFisher, AMF700) with a 20x objective. The entirety of each well was automatically scanned, resulting in 16 images from each well. Nuclei (Hoechst+) were identified using Stardist, and ACRV1+ cells were classified using CellProfiler. Hoechst+/ACRV1+ cells were counted as ACRV1+ cells and Hoechst+/ACRV1− cells were counted as ACRV1− cells, i.e., the other cells.

## 3. Results and Discussion

### 3.1. Spherical Bead Modeling

**Figure 3:**
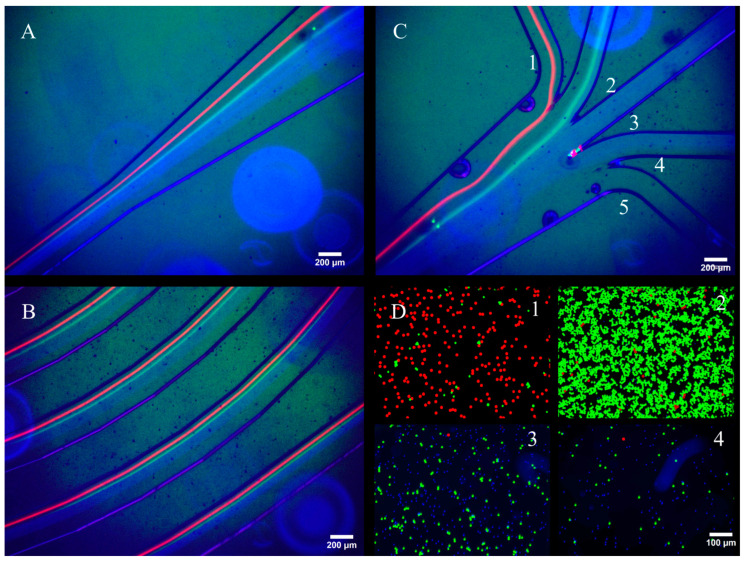
Validation of the model using spherical polystyrene beads. Fluorescent images during a test (**A**) right before the outlets split, (**B**) along the spiral turns, (**C**) at the outlet split, and (**D**) captured outputs from each outlet after the test. No beads were collected in outlet 5.

Figure 3 shows the images taken during the particle processing experiments right before the outlet split, along the spiral turns, and at the outlet junction. Figure 3A shows a visually distinctive separation between the 15 μm and 10 μm fluorescent particles, while the 6 μm particles had a wider band due to the smallest channel block ratio. Regarding focusing locations, the 15 μm particles focused on the innermost region of the spiral, followed by the 10 μm particles, while the 6 μm particles were distributed in the middle and the outer regions. The particles were also observed along the spiral turns, as shown in Figure 3B. The image shows that along the adjacent turns, the increased length resulted in better focusing of particles, thus reducing their linewidth along each outer turn. Figure 3C shows the particles right before the outlet split. Qualitatively, the 15 μm particles were extracted in outlet 1, 10 μm particles in outlet 2, and 6 μm particles were spread out among outlets 3 and 4. Lastly, Figure 3D shows the fluorescent images of the concentrated beads collected from the respective outlets for a qualitative analysis. Visually, outlet 1 captured most of the 15 μm particles, outlet 2 captured a significant amount of the 10 μm particles, and outlets 3 and 4 mostly captured the 6 μm particles; no beads were observed in outlet 5.

**Figure 4:**
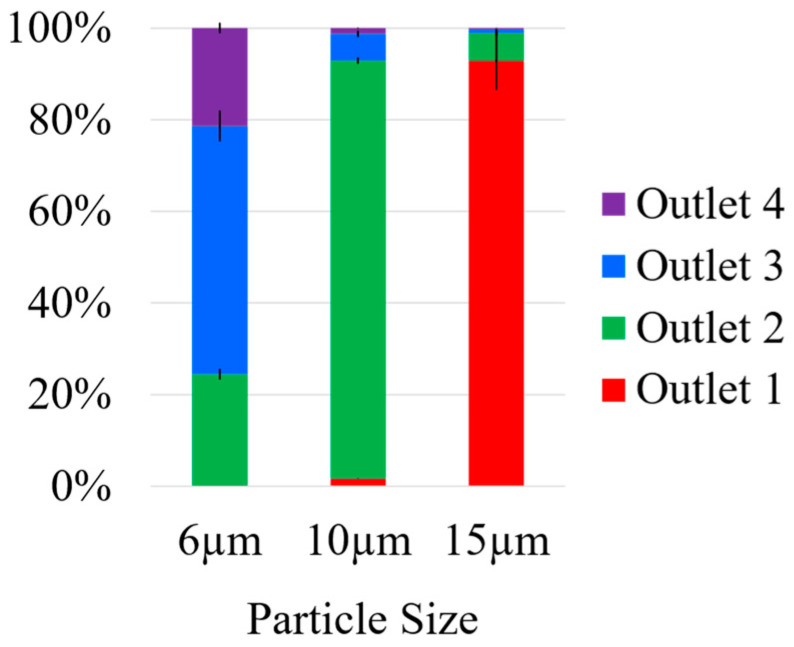
Size-based data from the image analysis of the captured spherical polystyrene beads from each outlet. Almost none of the 6 μm particles were captured in outlet 1, which captured most of the 15 μm size particles, thus isolating the larger particles. The smaller 6 μm particles were mostly focused to outlet 3, while being distributed almost evenly to outlets 2 and 4 as well. The 10 μm particles were mostly all captured in outlet 2.

Figure 4 shows a quantitative analysis, with the standard error of mean from triplicates, of the captured beads based on their size. In the case of the 6 μm particles, more than 50% were captured in outlet 3, while outlets 2 and 4 captured most of the remaining particles in equal amounts. This implies that the 6 μm particles were normally distributed among outlets 2, 3 and 4, with outlet 2 being the center of the focusing particle band. In the case of the 10 μm particles, more than 90% were captured in outlet 2, about 6% in outlet 3, and the remaining few distributed between outlets 1 and 4. Lastly, more than 90% of the 15 μm beads were captured in outlet 1, and about 6% in outlet 2. Only 0.2% of the beads captured in outlet 1 were 6 μm in size, thus isolating the 15 μm particles from the 6 μm.

In this relatively ideal case of highly spherical particles with generally uniform sizes, the device successfully removed the smallest 6 μm particles from the 15 μm particles. If required, a further optimized case would be one where the 6 μm particles could also result in a sharply focused band like the larger particles and yield an increased purity. Possible ways to achieve this would be to increase the channel block ratio for the particles that would benefit from a higher purity, i.e., decrease the channel height. However, upon reducing the channel height, the concentration of the particles would also need to be decreased to prevent defocusing because of an increased particle–particle interaction in the channel. Additionally, the flow rate in the channel could also be increased to achieve better particle focusing. However, this would also change the resulting secondary forces, which would need to be analyzed in tandem to find an appropriate balance between the two net forces, which could, in turn, result in different channel dimensions altogether. Ultimately, for our targeted purpose of removing particles closer to 6 μm in size, i.e., round spermatids, from larger particles, the device’s performance seemed appropriate based on the highly controlled test scenario of uniformly sized spheres.

### 3.2. Separation of the Spermatogenic Cells

#### 3.2.1. Rigid PMMA Device

**Figure 5:**
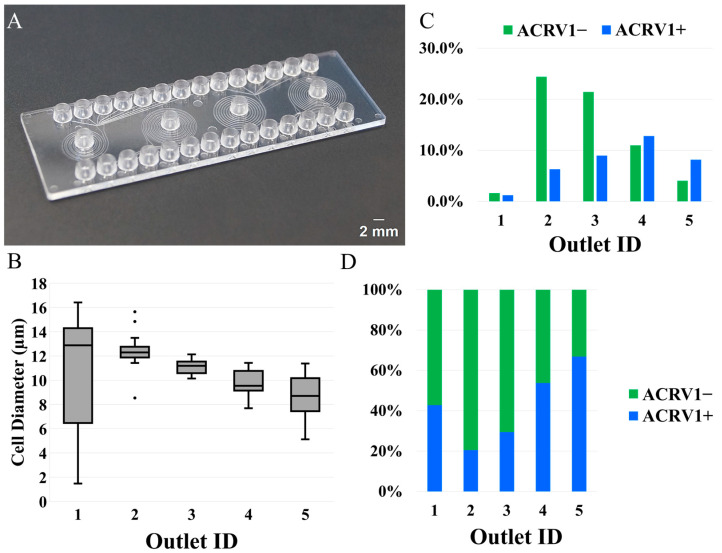
Cell sorting on the commercially available chip. (**A**) The commercially available microfluidic chip has eight outlets. The second spiral unit from the left was found to have parameters close to our requirements and was tested. (**B**) Size-based cell distribution among the outlets. Outlet 1 captured the widest range of cell sizes. The subsequent outlets captured a tighter range of cell sizes with decreasing average diameter. (**C**,**D**) ACRV1-based cell distribution along the outlets of the commercially available chip. Outlet 1 had the least amount of ACRV1+ contamination, but the recovery proportion was only 3% with almost an equal amount of ACRV1−. Outlets 2 and 3 recovered most of the ACRV1− with 21% and 30% of ACRV1+ contamination, respectively. Outlets toward the channel center, i.e., outlets 4 and 5, removed most of the remaining ACRV1+ contamination. Outlets 7 and 8 did not contain any cells.

Figure 5 shows the results obtained from the marker-based analysis of the captured outputs after running a sample containing a mix of testicular tissue cells. As discussed before, since the primary objective was to remove round spermatids from the middle outlets, the sample was treated with a round spermatid marker, ACRV1. Figure 5A shows the general-purpose, rigid, commercial device used in these experiments. An important note on this device is that the outlets were too narrow (90 μm each) for these specific cells and regularly got clogged after a few minutes, which had some impact on the data. Here, the innermost outlet is labeled as outlet 1, and the outer outlets are labeled with the subsequent numbers. Outlets 6, 7, and 8 are excluded since they were not supposed to and did not capture any cells. Figure 5B includes a box plot summarizing the size-based distribution among the outlets. As will be discussed later, outlet 1 captured very few cells with a wide range of cell sizes. The distribution was highly skewed, and the average cell size was around 13 μm. Outlet 2 captured a very tight range of cell sizes with a few outliers, and the average cell diameter was around 12 μm. The subsequent outlets captured cells with decreasing average cell sizes and slightly widening cell size ranges. As predicted, the outer outlets captured, on average, smaller cell sizes. Figure 5C plots the percentages of cells in each outlet compared to the total number of cells captured, and Figure 5D shows the cell composition from each outlet based on ACRV1. As predicted by the bead model, the outlet toward the channel center, i.e., outlet 4, removed most ACRV1+ cells. Outlet 1 resulted in the least amount of ACRV1+ contamination, but it also only captured an almost equal amount of ACRV1− cells. The plots, together with Table 2, show that outlet 1 recovered only 3% of the total captured cells, and 43% of those captured cells were ACRV1+, resulting in an ACRV1− purity of 57%. On the other hand, outlet 2 captured 31% of the total cells, and only 21% of the captured cells were ACRV1+ contamination, i.e., ACRV1− purity of 79%. Subsequent outlets captured 30%, 24%, and 12% of the total cells, and 30%, 54%, and 67%, respectively, of those captured cells were ACRV1+. Among the six outlets, outlets 3, 4, and 5 removed most of the ACRV1+ contamination from the sample, and outlets 2 and 3 recovered most of the ACRV1− cells. The 79% and 70% purity of ACRV1− captured in outlets 2 and 3, respectively, could be further improved by rerunning the cells through the device. In all cases, a substantial quantity of live cells was recovered providing significant quantities of live cells for further experiments.

#### 3.2.2. Flexible PDMS Device

**Figure 6:**
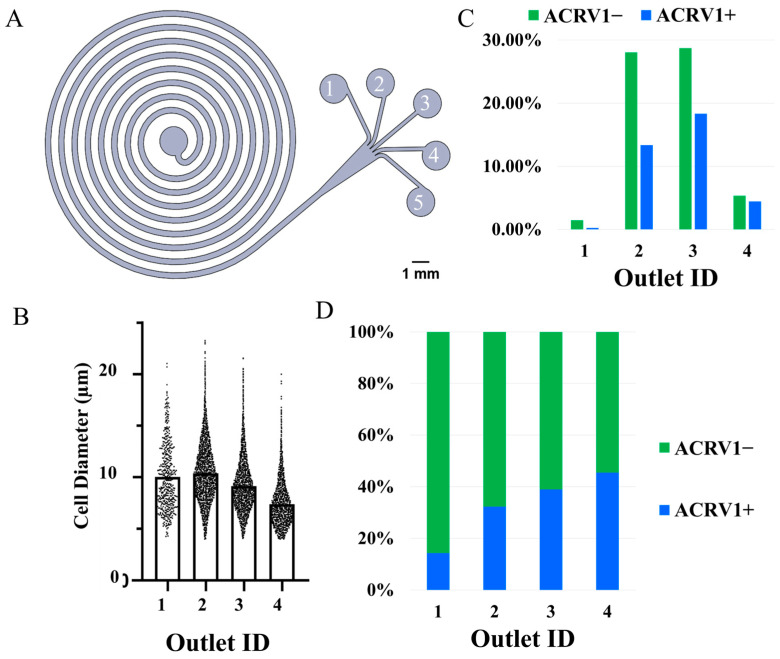
Size-based separation of the spermatogenic cells. (**A**) Device design and outlet labels. (**B**) Size-based cell distribution along the outlets. Outlet 1 captured the lowest number of cells with an average size of about 10 μm. Subsequent outlets captured cells in larger numbers and with lower mean diameters. On average, the largest mean size was around 10 μm, and the smallest mean size was around 7 μm. The sample contained very few cells in the range of 15 μm. (**C**,**D**) Distribution of the total cells captured among the four outlets. Outlet 1 included an almost negligible amount of ACRV1+ contamination. However, it also captured a minimal amount of ACRV1− cells since the sample originally contained very few cells close to 15 μm in size. Despite the low recovery, about 86% of cells captured in outlet 1 were ACRV1−, thus resulting in an increased purity. Outlets 2 and 3 removed most of the ACRV1+ contamination from the sample but also captured a significant amount of ACRV1− cells, respectively. This is explained by the mean sizes of ACRV1+ and ACRV1− in the sample being very close. The purity of ACRV1− cells collected in these outlets could be increased by rerunning the collected outputs.

The results obtained from the fabricated PDMS device are shown in Figure 6, where Figure 6A shows the labeling of the device outlets. One of the primary reasons for fabricating this device was to conform the outlet design to the calculated specifications. The outlets were made wider (200 μm each), and they were placed such that the fluid streamlines would be precisely split to capture specific sizes, as shown above in Figure 3C, and the device never resulted in any clogs. Figure 6B plots the size-based proportion of the captured cells in each outlet. Outlet 1 captured the least number of cells, and the mean cell diameter was around 10 μm, which could mean that the sample did not contain a lot of primary spermatocytes or that the primary spermatocytes were smaller in size. Qualitatively, outlets 2 and 3 captured more cells, and the size-based distribution of the cells also progressively skewed toward the lower end of the spectrum. The smallest mean cell diameter was around 7 μm in outlet 4. These plots show that the sample contained very few cells in the range of 15 μm and many cells with sizes ranging from 7 μm to 10 μm. Theoretically, this would result in a poorer resolution for the separated cells compared to the sample used in the commercial device. Figure 6C plots the percentage of ACRV1+ and ACRV1− cells captured in each outlet with reference to the total number of captured cells. With negligible ACRV1+ contamination, outlet 1 had the highest purity for the ACRV1− cells. Most ACRV1+ contamination was removed through outlets 2 and 3, but many ACRV1− cells were also captured in those outlets.

As can be inferred from Figure 6B, this low separation resolution is highly likely the result of minimal differences in the mean cell diameters among the outlets. Figure 6D details the composition of ACRV1+ and ACRV1− cells in each outlet. Only 14% of the captured cells in outlet 1 were ACRV1+, so this outlet provided increased purity of the ACRV1− cells. Cells captured in outlets 2, 3, and 4 comprised 32%, 39%, and 45% of the ACRV1+ cells, respectively. Since ACRV1+ marks the round spermatids whose average size is closer to 6 μm, the result follows a similar footprint made by the beads-based test model, where most of the 6 μm spherical beads were removed from the outlets toward the channel center. Outlet 2 recovered 41% of the total captured cells, with ACRV1+ contamination of only 31%. If deemed necessary, the ACRV1− from this outlet 2 could further be purified by rerunning the collected output through the device.

Table 3 lists the total number of cells counted from the well of each outlet, the resulting cell proportions compared to the total number of cells in the run, and the purity of ACRV1− among those captured cells. Outlet 1 recovered or captured 2% of the total cells captured among the outlets, and 86% of those captured cells were ACRV1−. The recovery is only 2% because this innermost outlet was designed to capture particles close to 15 μm. However, based on the size-based analysis, the sample contained very few cells in the range of 15 μm. Similarly, outlets 2, 3, and 4 captured 41%, 47%, and 10% of the total captured cells, and 68%, 61%, and 55%, respectively, of the cells captured in those outlets were ACRV1−. Of all the outlets, outlet 1 resulted in the best purification of ACRV1− cells. However, the best recovery with the lowest contamination resulted in outlet 2. For the best recovery and purity of ACRV1− cells, the captured output from outlet 2 could further be run through the device. In all cases, a substantial quantity of live cells was recovered providing significant quantities of live cells for further experiments.

The device design fabricated with PDMS and the commercial rigid device, combined, presented a high throughput separation method for the removal of the round spermatid contaminants from other cells of a spermatogenic cell sample. Compared to the fabricated PDMS device, which can flex during an experiment, the rigid device resulted in better particle behavior. However, unlike the general-purpose commercial device, since the outlets of the fabricated device were customized to the cells of interest, the device never resulted in clogs and still provided good quality results. If deemed necessary, the purity of ACRV1− could be further improved by performing multiple passes of the collected output through the device.

## 4. Conclusions

We successfully designed, tested, and demonstrated that a 300 μm × 80 μm spiral channel device with a radius of curvature of 7 mm can be used for the removal of human, round spermatids from other spermatogenic cells. Empirical knowledge based on the available literature helped us approximate the channel dimensions required to focus the 6 μm particle size. Additionally, a spherical bead-based model is still highly recommended to approximate different particle sizes’ focusing positions and line widths along the channel cross section. The bead results were highly repeatable and show the consistency of these types of devices. The device focused the smaller ACRV1+ cells (round spermatids) towards the channel center and increased the purity of ACRV1− cells collected through the channel’s inner outlets, thus validating the theoretical and experimental fluorescent bead model. In our experience, the conventional STA-PUT method was observed as resulting in a purity of 38%, while the reported device was able to purify cells other than round spermatids to 86%, a 126% improvement in purity. The device performs this separation in less than 10 min, while STA-PUT takes hours.

This microfluidic separation system only required a microfluidic device, a syringe pump, syringes, and tubes, thus resulting in a low-cost and compact cell-separation solution over a commercial STA-PUT apparatus. A commercial STA-PUT apparatus costs a lot more and requires a large temperature-controlled space to fit the apparatus since the cells are required to be within the system for hours. However, the cells spent less than 10 min in this microfluidic system (less than a few seconds in the spiral channel) and did not require any temperature control. With the advances in the resolution of 3D-printing techniques, these microfluidic devices could also be fabricated in-house at a much lower cost, thus making them even cheaper. One more benefit of using these small microfluidic devices over large systems is they can be multiplexed, thus making it possible to increase the overall throughput as desired.

One observed limitation of the design is the low ACRV1− recovery. However, since cells can always be cultured and increased in number, the low recovery is a lower priority compared to removing round spermatids from a sample. The round spermatids, i.e., the ACRV1+ contamination, present in a captured output could be further reduced by rerunning the captured cells through the microfluidic device.

Another major drawback of this technique is that it is very sensitive to disturbances in the flow. Since these microfluidic channels can be very small in cross sections, the channels are prone to becoming clogged, and more so in the case of cells which tend to adhere to each other and form larger blobs. If the flow in the channel becomes obstructed by some particles stuck in the channel or air bubbles trapped during the flow, the focused streams of particles can be disturbed. This disturbance can cause the focused cells or particles to defocus or even change the focusing locations, thus resulting in unintended outcomes. Additionally, the pressure drop along the channel length can be high in these spiral channel devices, especially at flow rates in the scale of ml/min. So, material strength and rigidity should also be carefully considered during the device design and operation.

Moreover, particle separation using inertial microfluidics is very sensitive to particle sizes. Cells, on the other hand, come in a range of sizes, thus making it difficult to align them into a sharp focused stream of cells. The result would be low purity and contamination of one cell-type into others. Thus, a device should always be designed while considering these characteristics. Ideally, we would want to design a system that focuses two different particle sizes as far away from each other as possible, thus reducing the contamination due to cell-size distribution.

During the experiments, the commercial device offered the benefit of consistency with its rigid channel dimensions but clogged repeatedly due to the outlets being too narrow and unoptimized for these cell types and their sizes. The fabricated PDMS design, on the other hand, provided the benefit of an optimized outlet design for the cells of interest, thus preventing any clogs in the device. Hence, future devices would be made from a rigid biocompatible material that precisely conforms to the channel and outlet design specifications calculated and discussed in this work. The device would, in principle, be able to separate these cells consistently while not resulting in clogs at the outlets.

Combined with the spherical bead-based experimental modeling, the theoretical model was successfully employed to design the required spiral channel, thus leading to an increased efficiency and throughput for separating round spermatids and other spermatogenic cells. The designed device can be fabricated at a low-cost and holds potential high-throughput applications not only in separating the spermatogenic cell phases but also in other cells and particles of sizes relevant to this study.

## Figures and Tables

**Table 1:** Variables and equations employed in the design of the spiral channel.

Equations	Variables
$D_{h}=\frac{2WH}{W+H}$	$D_{h}$: hydrodynamic diameter *W*: channel’s width *H*: channel’s height
$De={Re}_{c}\sqrt{\frac{D_{h}}{2r}}$	De: Dean number *r*: channel’s radius
${Re}_{c}=\rho \frac{uD_{h}}{\mu }$	${Re}_{c}$: channel’s Reynolds number u: flow velocity ρ: fluid density
$U_{D}=1.8\times 10^{-4}{De}^{1.63}$	$U_{D}$: Dean flow velocity
$\beta =\frac{a_{p}}{H}$	β: channel’s block ratio
$F_{L}=0.5\frac{a_{p}^{4}\rho U_{m}^{2}}{D_{h}^{2}}$	$F_{L}$: net inertial lift force $a_{p}$: particle size $U_{m}$: maximum flow velocity
$F_{D}=5.4\times 10^{-4}\pi \mu De^{1.63}a_{p}$	$F_{D}$: Dean drag force
$L_{f}=\frac{\pi µ}{\rho U_{m}\beta^{2}f_{L}}$	$L_{f}$: inertial focusing channel-length $f_{L}$: shear lift force coefficient

**Table 2:** Quantitative data obtained from the commercial device.

	Outlet 1	Outlet 2	Outlet 3	Outlet 4	Outlet 5
Total Cells	4119	44,431	43,962	34,412	17,665
Recovery %	3%	31%	30%	24%	12%
ACRV1− Purity	57%	79%	70%	46%	33%

**Table 3:** Quantitative data obtained from the fabricated device.

	Outlet 1	Outlet 2	Outlet 3	Outlet 4
Total Cells	509	12,214	13,876	2889
Recovery %	2%	41%	47%	10%
ACRV1− Purity	86%	68%	61%	55%

## Data Availability

The data that support the findings of this study are available from the corresponding author upon reasonable request.

## Supplementary Materials

The following supporting information can be downloaded at: https://www.mdpi.com/article/10.3390/mi16050500/s1, Figure S1: Fluorescent images of polystyrene beads with diameters of 6 μm (blue), 10 μm (green) and 15 μm (red) in channels of the same design but with different heights; Figure S2: Ratios of forces (*R_f_*) for 6, 10, and 15 μm particles plotted against different flow rates; Table S1: Characteristic parameters for the calculated design at a 1.5 mL/min flow rate.
