# Supplementary material for: Application of Inertial Microfluidics for Isolation and Removal of Round Spermatids from a Spermatogenic Cell Sample to Assist In-Vitro Human Spermatogenesis
Source: Micromachines (Basel). 2025 Apr 25;16(5):500. doi: 10.3390/mi16050500 (PMC12113712; doi:10.3390/mi16050500)
Supplement: Supplementary file 1 [file micromachines-16-00500-s001.zip › micromachines-3580398-supplementary.pdf]

Supplementary Material For:

Application of Inertial Microfluidics for Isolation and Removal of Round Spermatids from a Spermatogenic Cell Sample to Assist *In-Vitro* Human Spermatogenesis

Sabin Nepal [1], Joey Casalini [2], Alex Jafek [2] and Bruce Gale [1,2,*]

[1] Department of Mechanical Engineering, University of Utah, Salt Lake City, UT 84112, USA
[2] Paterna Biosciences Inc., Salt Lake City, UT 84119, USA
* Correspondence: bruce.gale@utah.edu

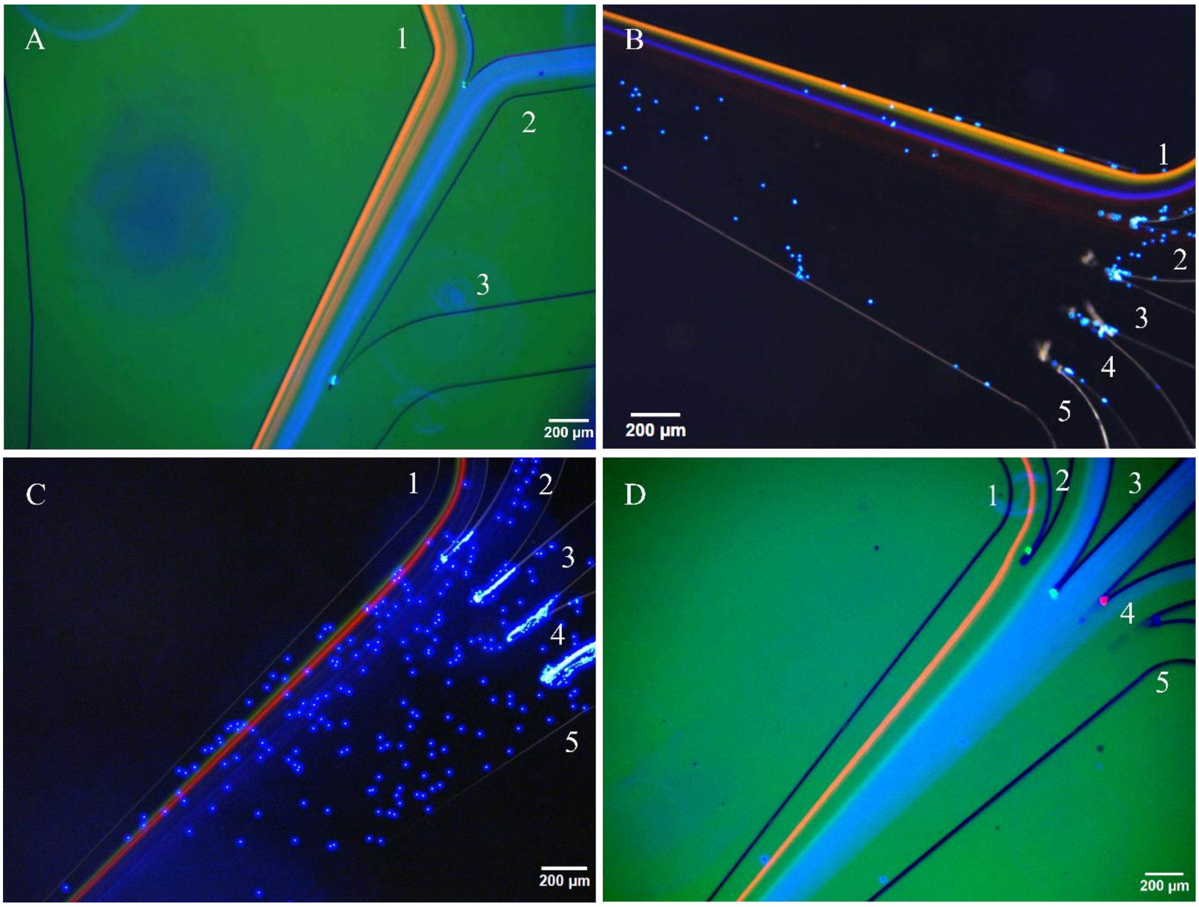

Figure S1. Fluorescent images of polystyrene beads with diameters of 6 µm (blue), 10 µm (green) and 15 µm (red) in channels of the same design but with different heights. (A) Channel cross section of 300 µm x 55 µm operated at 1 ml/min, but with a different outlet design. This design splits the channel into three outlets; 1 and 2 are 200 µm each and capture the inner 40% of the streamlines, while 3 is 600 µm and captures the outer 60% of the streamlines. The 55 µm height resulted in poor separation resolution between particles of different sizes, meaning insufficient Dean forces. (B) Channel cross section of 300 µm x 62 µm operated at 1 ml/min. Increasing the channel height at a constant flowrate decreases the inertial forces and increases the Dean drag forces. However, the increase in Dead drag was still insufficient to separate the particles at a higher resolution. (C) Channel cross section of 300 µm x 73 µm operated at 1 ml/min. At this height, we started seeing a better separation of the 6 µm particles from the larger particles. However, since the inertial forces drop with an increase in channel height, the smallest 6 µm particles were not sharply focused. Increase in Dean drag also exacerbates the issue by displacing the smallest particles sideways. (D) Channel cross section of 300 µm x 80 µm operated at 1 ml/min. The 6 µm particles reach the channel center, thus improving the particle separation resolution. However, a drawback of this displacement to the center is that the particles no longer focus sharply and result in a wider focusing band instead. A flow rate of 1.5 ml/min was finally selected for the final protocol to improve throughput while still maintaining the device's integrity during operation.

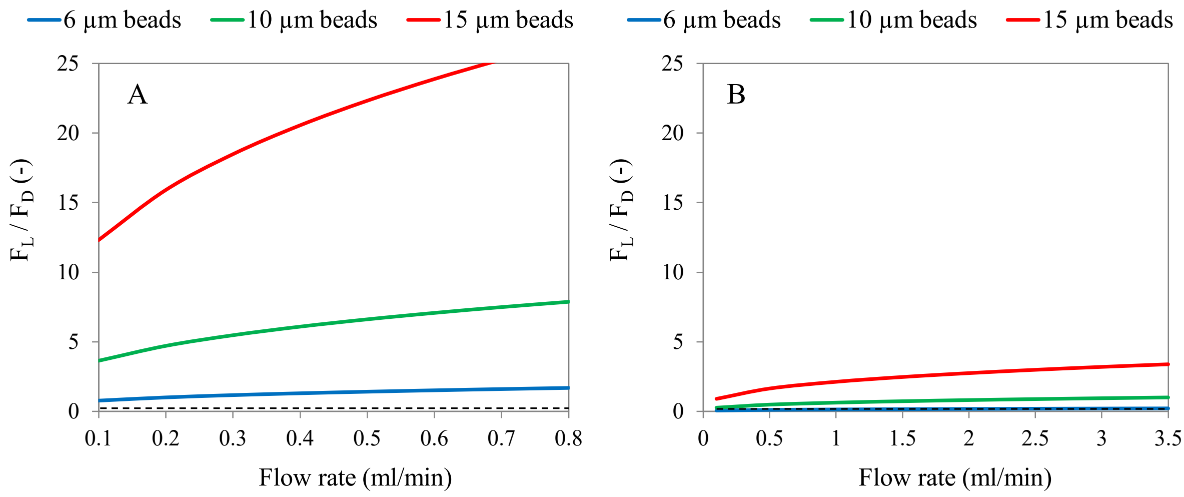

Figure S2. Ratios of forces (R_f) for 6, 10, and 15 µm particles plotted against different flow rates on the (A) 200 µm x 50 µm test spiral channel design and (B) 300 µm x 80 µm calculated spiral channel design. The dotted line ($R_f \sim 0.04 - 0.08$) represents an empirical threshold for R_f below which the Dean drag forces are significantly greater than the net inertial lift forces, thus tending to cause particle defocusing.

Table S1 Characteristic parameters for the calculated design at a 1.5 ml/min flow rate.

**Particle Size (µm)**	**Re**	**De**	**F_L (N)**	**F_D (N)**	**R_f**
**6**	131.5	12.5	9.92E-11	6.25E-10	0.16
**10**	131.5	12.5	7.65E-10	1.04E-09	0.73
**15**	131.5	12.5	3.87E-09	1.56E-09	2.48
